# Comparative genomic analysis of Streptococcus suis reveals significant genomic diversity among different serotypes

**DOI:** 10.1186/1471-2164-12-523

**Published:** 2011-10-25

**Authors:** Anding Zhang, Ming Yang, Pan Hu, Jiayan Wu, Bo Chen, Yafeng Hua, Jun Yu, Huanchun Chen, Jingfa Xiao, Meilin Jin

**Affiliations:** 1 State Key Laboratory of Agricultural Microbiology, Huazhong Agricultural University, Wuhan (430070), China; 2 CAS Key Laboratory of Genome Sciences and Information, Beijing Institute of Genomics, Chinese Academy of Sciences, Beijing(100029), China; 3 College of Veterinary Medicine, Huazhong Agricultural University, Wuhan (430070), China

## Abstract

**Background:**

*Streptococcus suis *(*S. suis*) is a major swine pathogen and an emerging zoonotic agent. Serotypes 1, 2, 3, 7, 9, 14 and 1/2 are the most prevalent serotypes of this pathogen. However, almost all studies were carried out on serotype 2 strains. Therefore, characterization of genomic features of other serotypes will be required to better understand their virulence potential and phylogenetic relationships among different serotypes.

**Results:**

Four Chinese *S. suis *strains belonging to serotypes 1, 7, 9 and 1/2 were sequenced using a rapid, high-throughput approach. Based on the 13 corresponding serotype strains, including 9 previously completed genomes of this bacterium, a full comparative genomic analysis was performed. The results provide evidence that (i) the pan-genome of this species is open and the size increases with addition of new sequenced genomes, (ii) strains of serotypes 1, 3, 7 and 9 are phylogenetically distinct from serotype 2 strains, but all serotype 2 strains, plus the serotype 1/2 and 14 strains, are very closely related. (iii) all these strains, except for the serotype 1 strain, could harbor a recombinant site for a pathogenic island (89 K) mediated by conjugal transfer, and may have the ability to gain the 89 K sequence.

**Conclusions:**

There is significant genomic diversity among different strains in *S. suis*, and the gain and loss of large amount of genes are involved in shaping their genomes. This is indicated by (i) pairwise gene content comparisons between every pair of these strains, (ii) the open pan-genome of this species, (iii) the observed indels, invertions and rearrangements in the collinearity analysis. Phylogenetic relationships may be associated with serotype, as serotype 2 strains are closely related and distinct from other serotypes like 1, 3, 7 and 9, but more strains need to be sequenced to confirm this.

## Background

*Streptococcus suis *(*S. suis*) is a major swine pathogen responsible for severe economic losses in the pork industry and is emerging as an important threat to human health, especially to people who have close contact with swine or pork by-products [1-3]. Since the first reported case of human meningitis caused by *S. suis *in Denmark in 1968, cases of infection have been reported continuously in more than 20 countries, with more than 700 people being affected [4]. Two recent large-scale outbreaks of human *S. suis *infections in China (one associated with 25 cases and 14 deaths in Jiangsu in 1998 and the other with 204 cases and 38 deaths in Sichuan in 2005) have raised awareness of the existing threat to public health [5-9]. The infection has also caused sporadic human illness in other countries, including Thailand [10-12], the United Kingdom [13], Portugal [14], Italy [15], Japan [16], Australia [17], the Netherlands [18] and the United States [19-22].

*S. suis *is an encapsulated Gram-positive coccus that possesses cell wall antigenic determinants, similar to Lancefield group D [23]. Among the 33 serotypes that have been classified based on the composition of their capsular polysaccharides (CPS), only a limited number are responsible for infections in pigs, including serotypes 1-9 and 14 [24]. Although the distribution of different serotypes varies depending on the geographical origins of the strains, *S. suis *serotype 2 (SS2) is considered the most pathogenic and the most prevalent capsular type among diseased pigs, followed by serotypes 3 and 1/2 [25,26]. Serotypes 1, 7 and 9 are also prevalent in several European [27,28] and Asian countries [26]. Serotype 14 infections in humans are now being reported with increasing frequency [29,30]. However, little information about these prevalent serotypes is available, except for serotype 2. Comparative genomic analysis is a powerful method for exploring the relationships between genotypes and phenotypes and for discovering genetic markers for clinical purposes.

A previous comparative genomic study based on examination of an intermediately pathogenic strain (89/1591), a highly pathogenic strain (GZ1) and an epidemic strain (SC84) indicates that acquiring particular genomic islands is essential for the evolution of highly pathogenic bacteria [9], and a specific pathogenic island (89 K) is found to be an essential component of virulent Chinese SS2 isolates [31,32]. A recent study indicates that the pathogenic island (89 K) can exhibit spontaneous excision to form an extrachromosomal circular product, which can then undergo lateral transfer to a recipient strain through site-specific recombination [33]. To understand the evolution of virulence in other prevalent serotypes, it is important to know whether they could also harbor recombinant target sites and serve as recipients for exogenous sequences.

In this study, we sequenced the genomes of 4 prevalent *S. suis *serotypes: 1, 1/2, 7 and 9. By taking the publicly available complete genome sequences of serotypes 2, 3 and 14 as the reference, a comparative genomic analysis was performed to provide a global genomic characterization of this prevalent pathogenic bacterium. Acquisitions and losses of genome components were identified, and different genes involved in CPS biosynthesis were found to be serotype determinants. The study also indicated that serotypes 1/2, 2, 3, 7 and 9, but not serotype 1, could supply a recombinant site for a pathogenic island (89 K) mediated by conjugal transfer, which suggests that these serotypes are able to obtain the 89 K sequence and thus become more virulent.

## Results and Discussion

### General features of the sequenced genomes

Among the 33 known serotypes, serotypes 1, 2, 3, 7, 9 and 1/2 are the most prevalent in pigs, and the strains causing human infections were also found among these serotypes [24-28]. Although 8 genome sequences of strains from serotype 2 were available, there was little information about the other serotypes, except for our recently updated genome sequences for serotypes 3 [34] and 14 [35]. In this study, whole genome sequencing was performed on 4 prevalent Chinese *S. suis *strains belonging to serotypes 1, 7, 9 and 1/2. Each of the 4 genomes was sequenced to a high level of redundancy (sequencing depth was 722 to 1627 fold). We filtered low-quality reads and used only high-quality reads for assembly. Reads for each genome were assembled into scaffolds, with 26 to 94 large scaffolds (>500bp) obtained per genome. Then scaffolds were aligned to the published genomes of *S. suis *to obtain linkage information for gap closure. All 4 annotated complete genomes were deposited in GenBank.

**Figure 1:**
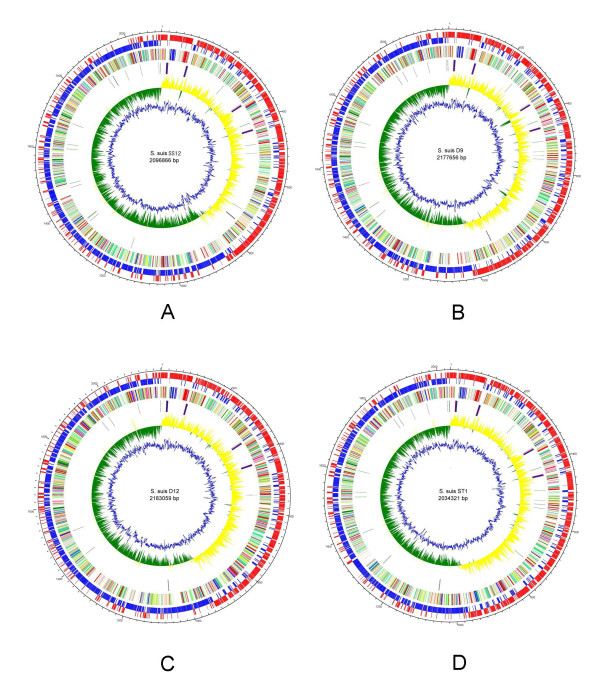
**Schematic circular diagrams of the S. suis SS12 (A), D9 (B), D12 (C) and ST1 genomes (D)**. Key for circular diagrams (outside to inside): scale (in kb); annotated CDSs are shown on a pair of concentric circles representing both coding strands (positive in red, negative in blue); COG functional classification, CDSs colored according to predicted functions; tRNAs and rRNAs, 16S rRNA displayed in red, 23S rRNA in blue, 5S rRNA in yellow, tRNA on positive strand in black, tRNA on negative strand in green; GC skew plot (>0% yellow, <0% green); G+C% content plot. Color coding for CDS functions: dark blue, pathogenicity/adaptation; black, energy metabolism; red, information transfer; dark green, surface-associated; cyan, degradation of large molecules; magenta, degradation of small molecules; yellow, central/intermediary metabolism; pale green, unknown; pale blue, regulators; orange, conserved hypothetical; brown, pseudogenes; pink, phage and IS elements; gray, miscellaneous.

Every genome consisted of a single circular chromosome with an approximate size of 2 Mb (Figure 1). Genome and assembly statistics for each strain were summarized in Table 1. The number of predicted ORFs for the 4 sequenced genomes was ranged from 2030 to 2136, and approximately 71% of the ORFs was assigned biological functions. The average gene length varied among different strains and was related to the number of pseudogenes and truncated genes presented. The genome of strain D9 (serotype 7) carried the greatest number of disrupted genes (126, or 5.9%), and conversely, SS12 (serotype 1/2) carried the lowest number (63, 3.0%) (Additional file 1). The average GC content was 41.2%, which was consistent with previous studies [8,31], and the genomic regions exhibiting an aberrant GC content may be the sites of horizontal gene transfer in different strains. Additionally, several IS elements were identified, and the number of IS elements was found to be similar to that of previously sequenced strains, such as P1/7, SC84 and BM407.

**Table 1:** General genome features and assembly statistics for each strain

**Sp**.	SS12	D9	D12	ST1
**Size (bp)**	2,096,866	2,177,656	2,183,059	2,034,321
**G+C %**	41.2	41.0	41.3	41.4
**CDSs**	2091	2136	2124	2030
**Coding %**	88.4	88.3	88.1	87.7
**Pseudogenes & Partial Genes**	63	126	100	100
**Avg. Gene Length (nt)**	887	901	906	879
**rRNA Locus**	4	4	4	4
**tRNA**	59	54	56	58
**IS Elements**	27	27	27	23
**Genome Islands**	16	16	23	6
**Coverage**	1042x	843x	722x	1627x
**Scaffold N50 (kb)**	130.4	37.8	76.9	44.2
**Scaffolds**	26	81	53	94

### Identification of gene clusters

**Figure 2:**
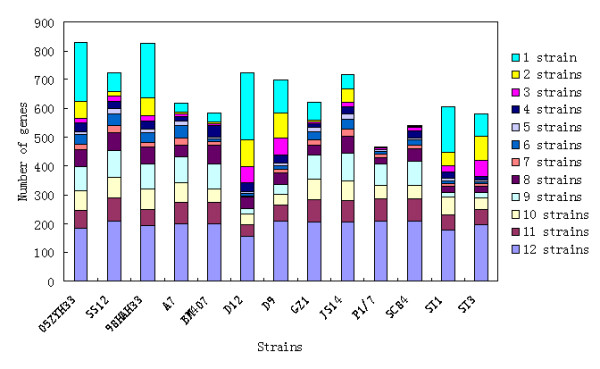
**Distribution of CDSs at different levels of conservation in each strain**. All noncore genes in each strain were classified into different levels of conservation according to the number of strains. Noncore genes present in 12 strains were considered the most conserved, whereas strain specific genes were the least conserved. Different conservations are represented by various colors.

**Figure 3:**
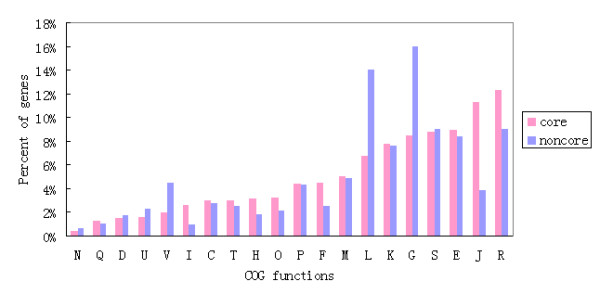
**Comparison of COG functional categories between core and noncore genes**. The ordinate axis indicates the gene percentage of a particular functional category relative to the genes of all COG categories. Letter coding for COG functions: C, energy production and conversion; D, Cell cycle control, cell division, chromosome partitioning; E, Amino acid transport and metabolism; F, Nucleotide transport and metabolism; G, Carbohydrate transport and metabolism; H, Coenzyme transport and metabolism; I, Lipid transport and metabolism; J, Translation, ribosomal structure and biogenesis; K, Transcription; L, Replication, recombination and repair; M, Cell wall/membrane/envelope biogenesis; N, Cell motility; O, Posttranslational modification, protein turnover, chaperones; P, Inorganic ion transport and metabolism; Q, Secondary metabolite biosynthesis, transport and catabolism; R, General function prediction only; S, Function unknown; T, Signal transduction mechanisms; U, Intracellular trafficking, secretion, and vesicular transport; V, Defense mechanisms.

All CDSs from the 13 completely sequenced *S. suis *genomes used for clustering were available in multi-FASTA format in the Supplemental Material (Additional file 2). There were 2374 *S. suis *orthologous gene clusters and 1211 unique genes, and the observed pan-genome shared by the 13 strains consisted of 3585 genes. The core genome of these strains comprised 1343 genes, accounting for 66.5% of total CDSs, and 28.9% of the genes were "dispensable" because they were shared by at least 2 strains, but not by all. All of the unique genes from these genomes only accounted for 4.6% of genes, but the percentage in each strain varied considerably (Figure 2). Non-core genes, including both dispensable genes and unique genes, usually play roles in nonessential metabolism and are more associated with virulence, environmental adaptation or serotype determination than core genes. Strain D9 possessed the highest proportion of non-core genes (38.6%), and strain P1/7 had the lowest proportion (26.4%). This may reflect different levels of gene gain and loss during the evolution of these strains or serotypes. Pairwise gene content comparisons among the 13 genomes indicated that the number of genes involved in gain and loss events between the strains was 587 on average. The largest number of gene difference between the strains was 1090, which was identified between strains D12 and 05ZYH33, and the minimum was 88, identified between strains P1/7 and SC84. A COG functional classification for core and non-core genes was performed, and the results showed that non-core genes were most likely to be assigned to categories, such as carbohydrate transport and metabolism, replication, recombination and repair, whereas core genes were more often associated with translation and ribosomal structure and biogenesis (Figure 3). Genes involved in translation, ribosomal structure and biogenesis lipid transport and metabolic functions were much less prevalent among non-core genes, while defense-related genes were more likely to be found among the core genes.

### Core and pan-genome analysis of *S. suis*

#### *S. suis *core genome

**Figure 4:**
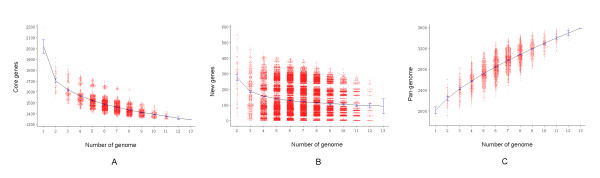
**Core and pan-genome calculations for S. suis**. **(A) *S. suis *core genome**. Each point represents the number of genes conserved between genomes. They are plotted as a function of strain numbers (x). For each x, the number of independent measurements is 13!/[n! (13-n)!]. The blue line demonstrates the exponential decay model based on the mean value of conserved genes. The curve is fitted to the function C(x) = Ac x^-tc ^+ yc. The best fit is obtained with correlation R2 = 0.993 for Ac = 880 ± 50, tc = 0.52 ± 0.06, yc = 1126 ± 55. (B) Decreasing number of new genes with sequential addition of new genomes. Numbers of new genes are calculated for all possible combinations and plotted as a function of strain numbers (x). The blue line demonstrates the exponential decay model based on the mean value of conserved genes. The curve is fitted to the function S(x) = As x-ts + ys. The best fit is obtained with correlation R2 = 0.995 for As = 489 ± 27, ts = 1.35 ± 0.09, ys = 82 ± 4. (C) *S. suis *pan-genome curve. The deduced pan-genome size P(x) = As (x-1) × ^-ts ^+ ys × - ys + Ac + yc. The curve continues to increase because the pan-genome of *S. suis *is open.

To determine the core genome of *S. suis*, the number of conserved genes found upon sequential addition of each new genome was extrapolated by fitting a decaying function that was considered to provide the best fit to the dataset (Figure 4A). Although the number of core genes initially decreased with the addition of each new genome, the core genome appeared to reach a plateau at approximately 1126 genes for *S. suis *species. The core gene number in each genome varied slightly because of the involvement of duplicated genes and paralogs in the shared clusters.

#### *S. suis *pan-genome analysis

To determine whether the *S. suis *pan-genome was open, the number of new genes (unique genes) was calculated every time a new genome was incorporated. As expected, the observed numbers varied greatly, as shown in Figure 4B. The large deviation from the mean suggested high levels of variation within *S. suis*. The mean values of new genes were used to perform the extrapolation. Similar to the core genes, the plot of new genes was fit well by a decaying function, and remarkably, the extrapolated curve reached an asymptotic value of 82, which meant that every newly sequenced genome could bring 82 new genes on average, even if many genomes were sequenced. This finding revealed that the species possesses an open pan-genome for which the size increases with the addition of new sequenced strains (Figure 4C). This was consistent with a previous study on the core and pan-genome of *Streptococcus*, which indicated that *S. suis *was the lineage with the largest number of gene gains and losses [36].

### Phylogenetic relationships among different serotype strains

**Figure 5:**
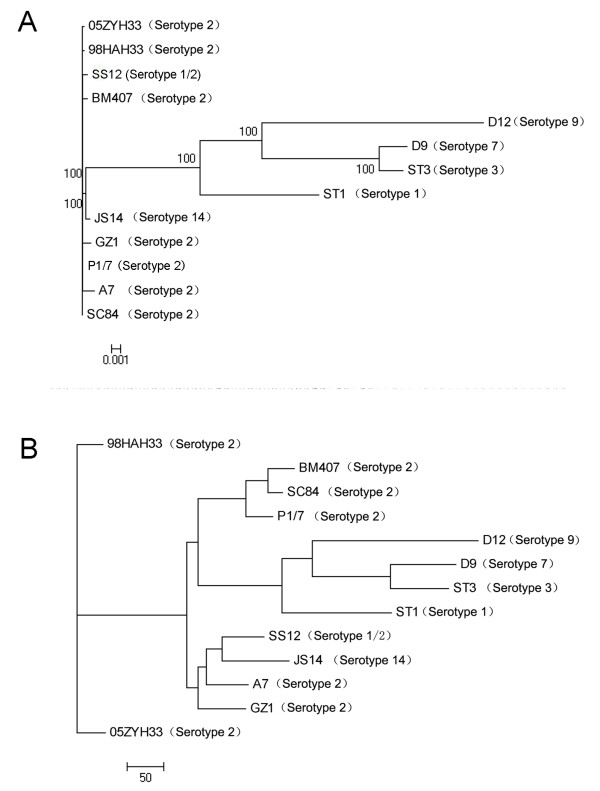
**Phylogenetic relationships of the 13 completely sequenced S. suis strains**. (A) Maximum likelihood tree based on 522 concatenated core genes with nearly identical lengths in the 13 *S. suis *genomes. Branch lengths indicate the number of SNPs, scale as indicated. (B) UPGMA (unweighted pair-group method with arithmetic means) tree based on the presence or absence of 2374 orthologous genes generated from clustering of all CDSs. All strain specific genes were removed to reduce the number of genes acquired occasionally during horizontal gene transfer. Branch lengths represent genetic differences occurring since the previous bifurcation node.

We used two methods to investigate the phylogenetic relationships among different serotypes, one of which was based on gene presence or absence among different strains, while the other one utilized the concatenated sequence of all single-copy core genes with exactly identical lengths from the 13 complete genomes. Figure 5A displays the phylogenetic relationships among the different strains based on the large sequence alignment of 522 core genes with the same length in each cluster. With the exception of serotype 14 strain JS14 and serotype 1/2 strain SS12, the non-serotype 2 strains appeared to be phylogenetically distinct from the serotype 2 strains, and could be assigned to a common clade. In this clade, serotype 7 strain D9 and serotype 3 strain ST3 were more closely related than any other pair. All serotype 2 strains presented an extremely short evolutionary distance from each other, indicating that these strains were probably derived from a recent common ancestor. It can also be inferred that phylogenetically serotype 1/2 and serotype 14 may be more closely related to the serotype 2 strains than the other 4 serotypes. However, to confirm this, more strains of other serotypes need to be sequenced. Figure 5B shows that the UPGMA (Unweighted Pair Group Method with Arithmetic Mean) phylogenetic tree reflect the number of gene gains and losses between all pairs of the 13 strains. The topologies of the MrBayes tree and the UPGMA tree bore some similarities. The 4 strains from serotypes 1, 3, 7 and 9 were also included in a common branch and differed greatly from the serotype 2 strains, whereas the serotype 1/2 and 14 strains were grouped into the same clade with A7 and GZ1 from serotype 2. The main difference between the two trees was that the two Chinese isolates, 05ZYH33 and 98HAH33, were more evolutionarily distant from the other serotype 2 strains indicated in the UPGMA tree.

### Genomic arrangement of *S. suis *strains

**Figure 6:**
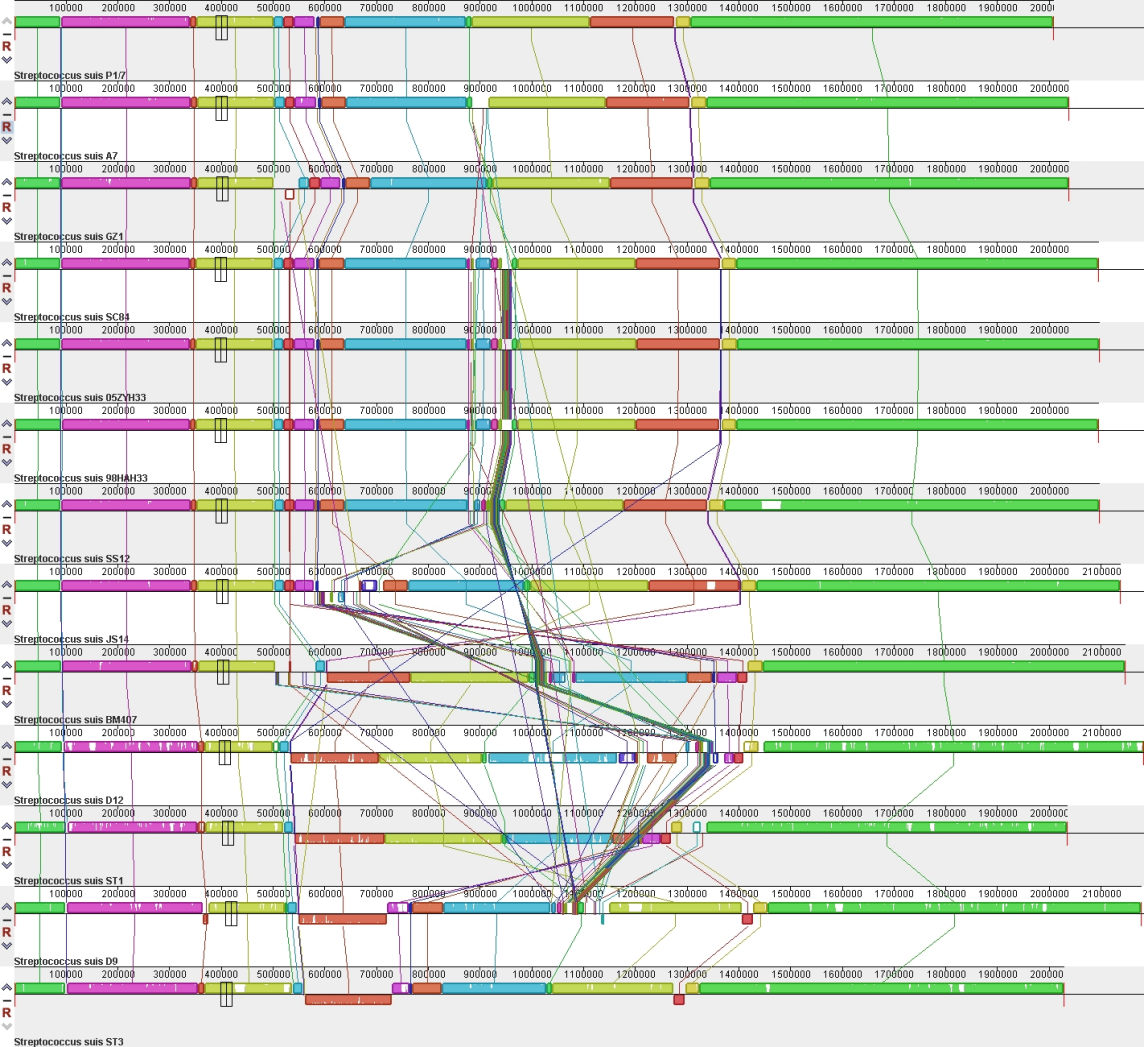
**Global multiple alignment of S. suis genomes**. The 13 genomes were compared to each other using progressive MAUVE with default parameters, and the collinearity of the genomes is shown. Sequence alignments that are free of rearrangements are shown as colored Local Collinear Blocks (LCBs). Sequence inversions are denoted by differential positioning of the LCBs relative to a reference axis.

A global multi-genome alignment of all 13 complete genomes was performed, and the results showed that some rearrangement occurred (Figure 6). These genomes could be classified into 3 categories according to their collinearity. All serotype 2 strains except for BM407, as well as the serotype 14 strain JS14 and serotype 1/2 strain SS12, were quite similar with respect to genome structure, with the exception of some small insertions. The genomes of BM407, D12 and ST1 shared a similar synteny with each other, and they displayed a large inversion when compared to that of other serotype 2 strains. The D9 and ST3 genomes were collinear along their length, with the exception of an insertion in the D9 genome. This is interesting because these synteny types were similar to some extent to the phylogenetic relationships seen among these strains.

### Genes involved in CPS biosynthesis

**Figure 7:**
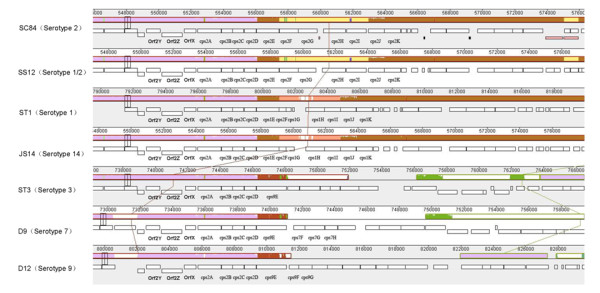
**Schematic comparison of the genetic organization of the CPS biosynthesis clusters in strains from different serotypes**. Among the serotype 2 strains, only S. suis SC84 is chosen for comparison, as the organization of the cps clusters among strains of this serotype is similar. The genomes of strains ST1 and D12 are inverted to provide a clear view, and the cps clusters included in them are on the negative strand. The comparison is shown using MAUVE. Annotated ORFs are indicated by outlined boxes.

*S. suis *is surrounded by a capsule that has been shown to be essential for its virulence [37,38]. It has been demonstrated that the presence of the capsule can decrease the activation of the PI-3K/Akt/PKCα signaling pathway involved in phagocytosis processes [39] and allows the bacterium to escape being killed by both macrophages and neutrophils [37,38,40]. The antigenic properties of capsular polysaccharides are the basis for serotype characterization. Only the structure of the serotype 2 capsular polysaccharide had previously been determined, and the genes involved in the biosynthesis of capsular polysaccharides had been found to be clustered in a single locus [41]. Orf2Y and Orf2Z are located upstream of the operon and may be involved in the regulation of these cps genes. Most of the serotypes include these two genes, as determined by hybrid assays [27], and the genome sequences indicate that these genes share very high sequence similarity. At the cps locus, the orfX and cpsA to cpsD genes could be hybridized in most serotypes [27], and these genes presented in all sequenced serotypes strains and highly conserved, indicating that they were involved in common functions related to the biosynthesis of the capsular polysaccharides, such as regulation, chain length determination and export, which were the functions of the homologous genes in *Streptococcus pneumoniae *[38]. The other 7 genes at the locus may be responsible for its specific CPS structure (Figure 7). The agglutination test indicated that the serotype 1/2 strains could react with hyperimmune sera against serotype 1 and serotype 2, and the sequenced genes encoding the CPS biosynthetic enzymes of serotype 1/2 showed high uniformity compared to serotype 2, suggesting that the modifications of the cps of serotype 1/2 were similar to those of serotype 1. Corresponding to the findings of a previous report [27], the genes coding for the CPS biosynthetic enzymes of serotypes 1 and 14 were highly conserved, indicating that the determinants for both serotypes include not only CPS structure, but also the modifications of polysaccharides (Figure 7).

### The prevalent serotypes supply a potential recombinant site for a pathogenic island (89 K)

The two large-scale outbreaks in China in 1998 and 2005 prompted researchers to determine which changes in the *S. suis *genome make it so highly virulent. Using comparative genomic analysis, an 89-kb sequence was identified only in the Chinese epidemic strain [31]. The subsequent investigation indicated that the 89-kb represented a GI-type T4SS-mediated horizontal transfer of a pathogenicity island that could be transferred to the recipient strain through a 15-bp sequence specific recombination event, although the transfer could be successfully observed only to serotype 2 [33]. Because the 89-kb harbored necessary elements for horizontal transfer, such as integrase, excisionase, DNA relaxase and so on, suggesting that this pathogenicity island maintained the potential to transfer to the recipient strain harboring the 15-bp sequence. The Genomic analysis indicated that the pathogenicity island did not exist in the other sequenced prevalent serotypes and such a 15-bp sequence could be found in the genomes of sequenced serotypes 1/2, 2, 3, 7 and 9, but not in serotype 1. More surprisingly, the flanking sequence structure of the 89 K region in the epidemic strain SC84 showed high similarity with the other sequenced serotypes, suggesting that these prevalent serotypes harboring the site for homologous recombination (the 15-bp sequence) would have the potential to act as recipient strains for the pathogenic island from the epidemic strain.

## Conclusion

In summary, comparative genomic analysis using genome sequences originating from prevalent *S. suis *serotypes showed that the observed pan genome of *S. suis *consists of 3585 gene clusters composed of 1343 core genome genes, 1031 distributed genes and 1211 strain-specific genes. The species possesses an open pan-genome and is the *Streptococcus *lineage with the greatest number of gene gains and losses. The results of this study also indicate that the other serotypes could supply a recombinant site for a pathogenic island (89 K) mediated by conjugal transfer, which suggests that these serotypes have the potential to obtain an 89 K sequence, and thus become more virulent. Our findings could be contributed to a better understanding of the genomics of *S. suis.*

## Methods

### Bacterial strains

Four Chinese isolated *S. suis *strains from the prevalent serotypes 1, 1/2, 7 and 9 were sequenced in this study. The characteristics of the sequenced strains and the publicly available genomes used for comparison are summarized in Table 2. The strains were maintained on tryptic soy agar (Difco Laboratories, Detroit) plus 10% bovine blood or cultured in Todd-Hewitt broth medium (Oxoid, Wesel, Germany) plus 10% bovine blood to mid-log phase (OD at 600 nm of 0.4) at 37°C under aerobic conditions. Total genomic DNA was extracted using the DNeasy Tissue Kit (Qiagen, Germany).

**Table 2:** Sequenced strains and genomes available in GenBank used in this study

Strain	Serotype	Place of origin	Plasmid	GenBank accession number	Reference
05ZYH33	2	China	no	CP000407	[31]
98HAH33	2	China	no	CP000408	[31]
A7	2	China	no	CP002570	Our other study ^a^
BM407	2	Vietnam	yes	FM252032/FM252033	[8]
D9	7	China	no	CP002641	This study
D12	9	China	no	CP002644	This study
GZ1	2	China	no	CP000837	[9]
JS14	14	China	no	CP002465	[35]
P1/7	2	Europe	no	AM946016	[8]
SC84	2	China	no	FM252031	[8]
SS12	1/2	China	no	CP002640	This study
ST1	1	China	no	CP002651	This study
ST3	3	China	no	CP002633	[34]

^a ^Strain A7 was sequenced in an unpublished study by our group.

### Sequencing and assembly

Bacterial genomes were sequenced at the Beijing Institute of Genomics (China) using a whole-genome shotgun sequencing strategy and Illumina Genome Analyzer sequencing technology. For each sample, a paired-end sequencing library containing fragments of approximate 500 bp was constructed. The short reads were filtered for quality and assembled with SOAPdenovo (http://soap.genomics.org.cn/soapdenovo.html). To fill the intra-scaffolds gaps, we used paired-end information to retrieve read pairs that had one read that was aligned to the contigs and another read that was located in the gap region. With this information, we did a local assembly for the collected reads. Then, these scaffolds were ordered relative to the genome of S. suis strain 05ZYH33 (deposited in the NCBI database; GenBank accession number CP000407) using MUMmer3 [42]. Gaps were closed by primer walking and sequencing of PCR products. Possible misassemblies were corrected using PCR amplification and direct sequencing. Sequences were edited in Consed [43].

### Genome annotation

Initially, Open Reading Frame (ORF) prediction was performed using Glimmer3 [44] and Genemarks [45], and the results were amalgamated. To avoid possible missing coding sequences, entire DNA sequences were compared to all known protein sequences from other published *S. suis *strains using BLAST searches. Then, all predicted ORFs were translated into amino acid sequences and compared against the non-redundant protein (nr) database using the BLASTp program, with a maximum expectation value of 1 × 10^-6^. ORFs with no BLAST hit to any other protein were automatically annotated as "hypothetical proteins." tRNAs and rRNAs were identified using tRNAscan-SE [46] and RNAmmer1.2 [47], respectively. Insertion sequence (IS) elements were found with IS Finder [48]. Genome islands (GIs) were identified using IslandViewer [49], which integrates three different genomic island prediction methods, followed by manual inspection.

The four annotated complete genome sequences have been deposited in GenBank with the accession numbers CP002640 (SS12), CP002641 (D9), CP002644 (D12) and CP002651 (ST1).

### Whole genome alignment and ortholog identification

Multiple genome alignments for 13 completely sequenced strains were constructed and visualized using the progressive Mauve program in Mauve v2.3.1 [50] at default settings.

All CDSs were extracted from the 13 *S. suis *genomes, and they were grouped into homologous clusters using InParanoid4 [51-53], which employs a BLAST reciprocal best hit algorithm, with default parameters.

### Core and pan-genome analysis

Tables of homologous clusters from InParanoid4 were compiled for identifying shared and unique genes. The numbers of conserved genes and unique genes depend on how many strains are taken into account. Thirteen strains with complete genome sequences were simulated in all possible combinations. The sizes of the core genome and novel gene set were calculated for each combination and then extrapolated using several functions to find a best fit from the mean number at each sampling point [54].

### Phylogenetic analysis

Phylogenetic trees of *S. suis *strains were constructed using two different methods [55]. The first utilized multiple sequence alignments of 522 single-copy core genes with nearly identical lengths and exactly one member in each of the compared strains. The alignments of these genes were concatenated into one large sequence alignment with a length of 457779 bp, and a phylogenetic tree was reconstructed using MrBayes 3 [56,57] (200,000 generations, sampled every 100 generations with a gamma distribution model and invariant class). The second method was based on the presence or absence of genes in the pan-genome. Genetic distances were defined as Σ _*n *_| *g*_*n, i *_- g_*n, k*_|, where *g*_*n, i *_is 1 if gene *n *is present in strain *i *and is zero otherwise. A dendrogram was generated using the UPGMA (unweighted pair group method with arithmetic mean) method implemented in the Phylip package [58].

## Authors' contributions

MJ and JX conceived the study; AZ, PH and MY annotated genomes, and performed analysis, AZ and PH wrote the manuscript; JW helped with the genome analysis; YH and BC identified and characterized the strains. HC and JY oversaw the genome sequencing and supervised the project. All authors read and approved the final manuscript.

## Supplementary Material

Additional file 1 **Pseudogenes and truncated genes in *S. suis *genomes**. Orthologues where present are presented in the same row. The systematic ID of mutated genes are indicated. Where orthologues in the other strains are not mutated they are listed as intact. Where orthologues are not present in the other strains they are listed as absent. Click here for file

Additional file 2 **All CDSs from the 13 completely sequenced *S. suis *genomes used for clustering**. This file is in FASTA format and can be viewed in any text editor. Click here for file

## References

[B1] Lun ZR Wang QP Chen XG Li AX Zhu XQ Streptococcus suis: an emerging zoonotic pathogen Lancet Infect Dis 2007 7 3 201 209 10.1016/S1473-3099(07)70001-4 17317601

[B2] Hill JE Gottschalk M Brousseau R Harel J Hemmingsen SM Goh SH Biochemical analysis, cpn60 and 16S rDNA sequence data indicate that Streptococcus suis serotypes 32 and 34, isolated from pigs, are Streptococcus orisratti Vet Microbiol 2005 107 1-2 63 69 10.1016/j.vetmic.2005.01.003 15795078

[B3] Staats JJ Feder I Okwumabua O Chengappa MM Streptococcus suis: past and present Vet Res Commun 1997 21 6 381 407 10.1023/A:1005870317757 9266659

[B4] Wertheim HF Nghia HD Taylor W Schultsz C Streptococcus suis: an emerging human pathogen Clin Infect Dis 2009 48 5 617 625 10.1086/596763 19191650

[B5] Tang J Wang C Feng Y Yang W Song H Chen Z Yu H Pan X Zhou X Wang H Streptococcal toxic shock syndrome caused by Streptococcus suis serotype 2 PLoS Med 2006 3 5 e151 10.1371/journal.pmed.0030151 16584289 PMC1434494

[B6] Yu H Jing H Chen Z Zheng H Zhu X Wang H Wang S Liu L Zu R Luo L Human Streptococcus suis outbreak, Sichuan, China Emerg Infect Dis 2006 12 6 914 920 16707046 10.3201/eid1206.051194 PMC3373052

[B7] Segura M Streptococcus suis: An Emerging Human Threat J Infect Dis 2009 199 1 4 6 10.1086/594371 19016626

[B8] Holden MT Hauser H Sanders M Ngo TH Cherevach I Cronin A Goodhead I Mungall K Quail MA Price C Rapid evolution of virulence and drug resistance in the emerging zoonotic pathogen Streptococcus suis PLoS One 2009 4 7 e6072 10.1371/journal.pone.0006072 19603075 PMC2705793

[B9] Ye C Zheng H Zhang J Jing H Wang L Xiong Y Wang W Zhou Z Sun Q Luo X Clinical, Experimental, and Genomic Differences between Intermediately Pathogenic, Highly Pathogenic, and Epidemic Streptococcus suis J Infect Dis 2009 199 1 97 107 10.1086/594370 19016627

[B10] Wangsomboonsiri W Luksananun T Saksornchai S Ketwong K Sungkanuparph S Streptococcus suis infection and risk factors for mortality J Infect 2008 57 5 392 396 10.1016/j.jinf.2008.08.006 18835496

[B11] Rusmeechan S Sribusara P Streptococcus suis meningitis: the newest serious infectious disease J Med Assoc Thai 2008 91 5 654 658 18672627

[B12] Takamatsu D Wongsawan K Osaki M Nishino H Ishiji T Tharavichitkul P Khantawa B Fongcom A Takai S Sekizaki T Streptococcus suis in humans, Thailand Emerg Infect Dis 2008 14 1 181 183 10.3201/eid1401.070568 18258106 PMC2600138

[B13] Watkins EJ Brooksby P Schweiger MS Enright SM Septicaemia in a pig-farm worker Lancet 2001 357 9249 38 10.1016/S0140-6736(00)03570-4 11197360

[B14] Taipa R Lopes V Magalhaes M Streptococcus suis meningitis: first case report from Portugal J Infect 2008 56 6 482 483 10.1016/j.jinf.2008.03.002 18433873

[B15] Manzin A Palmieri C Serra C Saddi B Princivalli MS Loi G Angioni G Tiddia F Varaldo PE Facinelli B Streptococcus suis meningitis without history of animal contact, Italy Emerg Infect Dis 2008 14 12 1946 1948 10.3201/eid1412.080679 19046529 PMC2634631

[B16] Chang B Wada A Ikebe T Ohnishi M Mita K Endo M Matsuo H Asatuma Y Kuramoto S Sekiguchi H Characteristics of Streptococcus suis isolated from patients in Japan Jpn J Infect Dis 2006 59 6 397 399 17186962

[B17] Tramontana AR Graham M Sinickas V Bak N An Australian case of Streptococcus suis toxic shock syndrome associated with occupational exposure to animal carcasses Med J Aust 2008 188 9 538 539 18459929 10.5694/j.1326-5377.2008.tb01771.x

[B18] van de Beek D Spanjaard L de Gans J Streptococcus suis meningitis in the Netherlands J Infect 2008 57 2 158 161 10.1016/j.jinf.2008.04.009 18538852

[B19] Smith TC Capuano AW Boese B Myers KP Gray GC Exposure to Streptococcus suis among US swine workers Emerg Infect Dis 2008 14 12 1925 1927 10.3201/eid1412.080162 19046523 PMC2634616

[B20] Fittipaldi N Collis T Prothero B Gottschalk M Streptococcus suis Meningitis, Hawaii Emerg Infect Dis 2009 15 12 2067 2069 19961708 10.3201/eid1512.090825 PMC3044538

[B21] Lee GT Chiu CY Haller BL Denn PM Hall CS Gerberding JL Streptococcus suis meningitis, United States Emerg Infect Dis 2008 14 1 183 185 10.3201/eid1401.070930 18258107 PMC2600143

[B22] Willenburg KS Sentochnik DE Zadoks RN Human Streptococcus suis meningitis in the United States N Engl J Med 2006 354 12 1325 10.1056/NEJMc053089 16554543

[B23] Gottschalk M Xu J Calzas C Segura M Streptococcus suis: a new emerging or an old neglected zoonotic pathogen? Future Microbiol 2010 5 371 391 10.2217/fmb.10.2 20210549

[B24] Gottschalk M Segura M Xu J Streptococcus suis infections in humans: the Chinese experience and the situation in North America Anim Health Res Rev 2007 8 1 29 45 10.1017/S1466252307001247 17692141

[B25] Messier S Lacouture S Gottschalk M Distribution of Streptococcus suis capsular types from 2001 to 2007 Can Vet J 2008 49 5 461 462 18512456 PMC2359489

[B26] Wei Z Li R Zhang A He H Hua Y Xia J Cai X Chen H Jin M Characterization of Streptococcus suis isolates from the diseased pigs in China between 2003 and 2007 Vet Microbiol 2009 137 1-2 196 201 10.1016/j.vetmic.2008.12.015 19185432

[B27] Smith HE Veenbergen V van der Velde J Damman M Wisselink HJ Smits MA The cps genes of Streptococcus suis serotypes 1, 2, and 9: development of rapid serotype-specific PCR assays J Clin Microbiol 1999 37 10 3146 3152 10488168 10.1128/jcm.37.10.3146-3152.1999 PMC85514

[B28] Smith HE van Bruijnsvoort L Buijs H Wisselink HJ Smits MA Rapid PCR test for Streptococcus suis serotype 7 FEMS Microbiol Lett 1999 178 2 265 270 10.1111/j.1574-6968.1999.tb08686.x 10499276

[B29] Haleis A Alfa M Gottschalk M Bernard K Ronald A Manickam K Meningitis caused by Streptococcus suis serotype 14, North America Emerg Infect Dis 2009 15 2 350 352 10.3201/eid1502.080842 19193296 PMC2657623

[B30] Poggenborg R Gaini S Kjaeldgaard P Christensen JJ Streptococcus suis: meningitis, spondylodiscitis and bacteraemia with a serotype 14 strain Scand J Infect Dis 2008 40 4 346 349 10.1080/00365540701716825 18365920

[B31] Chen C Tang J Dong W Wang C Feng Y Wang J Zheng F Pan X Liu D Li M A glimpse of streptococcal toxic shock syndrome from comparative genomics of S. suis 2 Chinese isolates PLoS ONE 2007 2 3 e315 10.1371/journal.pone.0000315 17375201 PMC1820848

[B32] Li M Wang C Feng Y Pan X Cheng G Wang J Ge J Zheng F Cao M Dong Y SalK/SalR, a two-component signal transduction system, is essential for full virulence of highly invasive Streptococcus suis serotype 2 PLoS ONE 2008 3 5 e2080 10.1371/journal.pone.0002080 18461172 PMC2358977

[B33] Li M Shen X Yan J Han H Zheng B Liu D Cheng H Zhao Y Rao X Wang C GI-type T4SS-mediated horizontal transfer of the 89K pathogenicity island in epidemic Streptococcus suis serotype 2 Mol Microbiol 2011 10.1111/j.1365-2958.2011.07553.x PMC3132442 21244532

[B34] Hu P Yang M Zhang A Wu J Chen B Hua Y Yu J Chen H Xiao J Jin M Complete genome sequence of Streptococcus suis serotype 3 strain ST3 J Bacteriol 2011 193 13 3428 3429 10.1128/JB.05018-11 21572001 PMC3133292

[B35] Hu P Yang M Zhang A Wu J Chen B Hua Y Yu J Xiao J Jin M Complete Genome Sequence of Streptococcus suis Serotype 14 Strain JS14 J Bacteriol 193 9 2375 2376 10.1128/JB.00083-11 PMC3133069 21398551

[B36] Lefebure T Stanhope MJ Evolution of the core and pan-genome of Streptococcus: positive selection, recombination, and genome composition Genome Biol 2007 8 5 R71 10.1186/gb-2007-8-5-r71 17475002 PMC1929146

[B37] Charland N Harel J Kobisch M Lacasse S Gottschalk M Streptococcus suis serotype 2 mutants deficient in capsular expression Microbiology 1998 144 Pt 2 325 332 9493370 10.1099/00221287-144-2-325

[B38] Smith HE Damman M van der Velde J Wagenaar F Wisselink HJ Stockhofe-Zurwieden N Smits MA Identification and characterization of the cps locus of Streptococcus suis serotype 2: the capsule protects against phagocytosis and is an important virulence factor Infect Immun 1999 67 4 1750 1756 10085014 10.1128/iai.67.4.1750-1756.1999 PMC96524

[B39] Segura M Gottschalk M Olivier M Encapsulated Streptococcus suis inhibits activation of signaling pathways involved in phagocytosis Infect Immun 2004 72 9 5322 5330 10.1128/IAI.72.9.5322-5330.2004 15322029 PMC517481

[B40] Chabot-Roy G Willson P Segura M Lacouture S Gottschalk M Phagocytosis and killing of Streptococcus suis by porcine neutrophils Microb Pathog 2006 41 1 21 32 10.1016/j.micpath.2006.04.001 16714092

[B41] Van Calsteren MR Gagnon F Lacouture S Fittipaldi N Gottschalk M Structure determination of Streptococcus suis serotype 2 capsular polysaccharide Biochem Cell Biol 2010 88 3 513 525 10.1139/O09-170 20555393

[B42] Kurtz S Phillippy A Delcher AL Smoot M Shumway M Antonescu C Salzberg SL Versatile and open software for comparing large genomes Genome Biol 2004 5 2 R12 10.1186/gb-2004-5-2-r12 14759262 PMC395750

[B43] Gordon D Abajian C Green P Consed: a graphical tool for sequence finishing Genome Res 1998 8 3 195 202 9521923 10.1101/gr.8.3.195

[B44] Delcher AL Harmon D Kasif S White O Salzberg SL Improved microbial gene identification with GLIMMER Nucleic Acids Res 1999 27 23 4636 4641 10.1093/nar/27.23.4636 10556321 PMC148753

[B45] Besemer J Borodovsky M GeneMark: web software for gene finding in prokaryotes, eukaryotes and viruses Nucleic Acids Res 2005 33 Web Server W451 454 10.1093/nar/gki487 15980510 PMC1160247

[B46] Lowe TM Eddy SR tRNAscan-SE: a program for improved detection of transfer RNA genes in genomic sequence Nucleic Acids Res 1997 25 5 955 964 10.1093/nar/25.5.955 9023104 PMC146525

[B47] Lagesen K Hallin P Rodland EA Staerfeldt HH Rognes T Ussery DW RNAmmer: consistent and rapid annotation of ribosomal RNA genes Nucleic Acids Res 2007 35 9 3100 3108 10.1093/nar/gkm160 17452365 PMC1888812

[B48] Siguier P Perochon J Lestrade L Mahillon J Chandler M ISfinder: the reference centre for bacterial insertion sequences Nucleic Acids Res 2006 34 Database D32 36 16381877 10.1093/nar/gkj014 PMC1347377

[B49] Langille MG Brinkman FS IslandViewer: an integrated interface for computational identification and visualization of genomic islands Bioinformatics 2009 25 5 664 665 10.1093/bioinformatics/btp030 19151094 PMC2647836

[B50] Darling AE Mau B Perna NT progressiveMauve: multiple genome alignment with gene gain, loss and rearrangement PLoS One 5 6 e11147 10.1371/journal.pone.0011147 PMC2892488 20593022

[B51] Remm M Storm CE Sonnhammer EL Automatic clustering of orthologs and in-paralogs from pairwise species comparisons J Mol Biol 2001 314 5 1041 1052 10.1006/jmbi.2000.5197 11743721

[B52] O'Brien KP Remm M Sonnhammer EL Inparanoid: a comprehensive database of eukaryotic orthologs Nucleic Acids Res 2005 33 Database D476 480 15608241 10.1093/nar/gki107 PMC540061

[B53] Alexeyenko A Tamas I Liu G Sonnhammer EL Automatic clustering of orthologs and inparalogs shared by multiple proteomes Bioinformatics 2006 22 14 e9 15 10.1093/bioinformatics/btl213 16873526

[B54] Donati C Hiller NL Tettelin H Muzzi A Croucher NJ Angiuoli SV Oggioni M Dunning Hotopp JC Hu FZ Riley DR Structure and dynamics of the pan-genome of Streptococcus pneumoniae and closely related species Genome Biol 2010 11 10 R107 10.1186/gb-2010-11-10-r107 21034474 PMC3218663

[B55] Wilcox TP Zwickl DJ Heath TA Hillis DM Phylogenetic relationships of the dwarf boas and a comparison of Bayesian and bootstrap measures of phylogenetic support Mol Phylogenet Evol 2002 25 2 361 371 10.1016/S1055-7903(02)00244-0 12414316

[B56] Ronquist F Huelsenbeck JP MrBayes 3: Bayesian phylogenetic inference under mixed models Bioinformatics 2003 19 12 1572 1574 10.1093/bioinformatics/btg180 12912839

[B57] Huelsenbeck JP Ronquist F MRBAYES: Bayesian inference of phylogenetic trees Bioinformatics 2001 17 8 754 755 10.1093/bioinformatics/17.8.754 11524383

[B58] Baum BR PHYLIP: Phylogeny Inference Package. Version 3.2. (Software review) Quarterly Review of Biology 1989 64 539 541 10.1086/416571

